# Graphene-Derived Materials Interfacing the Spinal Cord: Outstanding *in Vitro* and *in Vivo* Findings

**DOI:** 10.3389/fnsys.2017.00071

**Published:** 2017-09-27

**Authors:** Ana Domínguez-Bajo, Ankor González-Mayorga, Elisa López-Dolado, María C. Serrano

**Affiliations:** ^1^Laboratory of Interfaces for Neural Repair, Hospital Nacional de Parapléjicos, Servicio de Salud de Castilla-La Mancha, Toledo, Spain; ^2^Research Unit of “Design and development of biomaterials for neural regeneration”, Hospital Nacional de Parapléjicos, Servicio de Salud de Castilla-La Mancha, Joint Research Unit with CSIC, Toledo, Spain; ^3^Laboratory of Materials for Health, Instituto de Ciencia de Materiales de Madrid, Consejo Superior de Investigaciones Científicas, Madrid, Spain; ^4^Materials Science Factory, Instituto de Ciencia de Materiales de Madrid, Consejo Superior de Investigaciones Científicas, Madrid, Spain

**Keywords:** graphene, spinal cord injuries, scaffold, toxicity, neurons, *in vivo* models

## Abstract

The attractiveness of graphene-derived materials (GDMs) for neural applications has fueled their exploration as components of biomaterial interfaces contacting the brain and the spinal cord. In the last years, an increasing body of work has been published on the ability of these materials to create biocompatible and biofunctional substrates able to promote the growth and activity of neural cells *in vitro* and positively interact with neural tissues when implanted *in vivo*. Encouraging results in the central nervous tissue might impulse the study of GDMs towards preclinical arena. In this mini-review article, we revise the most relevant literature on the interaction of GDMs with the spinal cord. Studies involving the implantation of these materials *in vivo* in the injured spinal cord are first discussed, followed by models with spinal cord slides *ex vivo* and a final description of selected results with neural cells *in vitro*. A closing debate of the major conclusions of these results is presented to boost the investigation of GDMs in the field.

## Introduction

The outstanding physico-chemical properties of carbon-based materials including carbon nanotubes (CNTs), carbon nanofibers and graphene are encouraging their exploration as attractive materials to interface the damaged neural tissue as part of novel platforms with potential utility in neural repair (Tavangarian and Li, 2012). One of the main properties of these materials is their ability to adsorb molecules. This fact brings high versatility for biological applications due to the possibility of modifying their surface characteristics to induce and modulate specific cell and tissue responses. Specifically, graphene displays a large surface area enhancing adsorptive features (Li et al., 2008). Graphene oxide (GO) presents a higher ability to adsorb molecules as a consequence of the presence of oxygen-containing chemical groups that serve attracting adhesive moieties, cell media components and therapeutic drugs (Yang et al., 2008). Moreover, GO is more hydrophilic than pristine graphene, thus increasing dispersibility and diminishing aggregation. An additional feature that make graphene-derived materials (GDMs) attractive for interfacing the injured central nervous tissue is their capacity to cross the blood-brain and blood-spinal cord barriers by using specific functionalization protocols (Yang et al., 2015). Other remarkable properties, such as their mechanical behavior (Lee et al., 2008), allow the preparation of flexible 3D structures which are mechanically compliant with neural tissues. Finally, their superior charge carrier mobility (Soldano et al., 2010) is of interest for electrical stimulation and recording in neural tissues and cells.

This mini-review article focuses mainly on the most relevant and recent publications to date on the exploration of GDMs interfacing the spinal cord, including *in vivo*, *ex vivo* and *in vitro* models. Outstanding work of GDMs implanted in the brain is also discussed. Major findings discussed in this mini-review article are summarized in Table 1. Readers are referred to excellent reviews in the topic for further details (Fattahi et al., 2014; Fraczek-Szczypta, 2014; Nakanishi et al., 2014; John et al., 2015).

**Table 1:** Summary of major findings in the exploration of graphene-derived materials (GDMs) interfacing spinal cord components.

Material	Shape	Configuration	Model	Study system	Major findings	Reference
GO	Nanosheets	2D	*In vivo*	Zebrafish embryo	Hypoxic environment near the embryo Concentration-dependent tail/spinal cord flexure	Chen et al. (2016)
RGO	Nanosheets	2D	*In vivo*	Olfactory bulb of mice	Survival of resident cell populations Preservation of *de novo* neurogenesis	Defterali et al. (2016)
RGO	Porous scaffolds	3D	*In vivo*	Spinal cord hemisection at C6 in rats	Formation of soft interface with neural tissue No augmentation of fibroglial scars Cellular and molecular scaffold infiltration Abundance of vimentin^+^ and PDGFRβ^+^ cells Presence of M2-like macrophages	López-Dolado et al. (2015)
RGO	Porous scaffolds	3D	*In vivo*	Spinal cord hemisection at C6 in rats	Enhanced collagen scaffold infiltration Reduction of vimentin^+^ and ED1^+^ cells Augmentation of M2-like macrophages Angiogenesis inside scaffold New axons formed inside scaffold	López-Dolado et al. (2016)
RGO	Poorly and highly magnetic scaffolds	3D	*In vivo*	Intrathecal injection in subarachnoid space in rats	More intense nociceptive responses induced by highly magnetic RGO Alteration of voltage-gated Ca^2+^ channels Alteration of NMDA receptors	Manzo et al. (2017)
RGOPEG	Nanosheets	Liquid	*In vitro* *In vivo*	Astrocytes and endothelial cells Intravenous injection	Dose and time-dependent toxicity Downregulation of tight junction proteins Increase in oxidative stress proteins	Mendonça et al. (2016)
Graphene	Flakes	2D	*In vivo*	Fertilized chicken eggs	Alterations in brain structure Chicken survival significantly decreased	Sawosz et al. (2014)
MWCNTs	Scaffolds	3D	*Ex vivo*	Spinal cord slices	Formation of functional electrical connections between slices through MWCNT scaffolds	Usmani et al. (2016)
MWCNTs	Scaffolds	3D	*In vitro*	Dissociated spinal cord neurons	Anticipation of neuron maturation Modulation of gene expression Triggering of microglia reparative responses	Fabbro et al. (2013)
GO	Small and large nanosheets	2D	*In vitro*	Primary hippocampal neurons Cortical glial cultures	Reduction of frequency of spontaneous post-synaptic currents, increase of inter-event intervals in Ca^2+^ oscillations and decrease in VGLUT1 levels induced by small-GO	Rauti et al. (2016)

*Abbreviations: GO, graphene oxide; MWCNTs, multi-walled carbon nanotubes; NMDA, N-methyl-D-aspartate; PDGFRβ, β receptor of the platelet-derived growth factor; PEG, poly(ethylene-glycol); RGO, reduced graphene oxide; VGLUT1, vesicular glutamate transporter 1*.

## Spinal Cord Injury and its Physiopathology

Human spinal cord injuries (SCIs) affect thousands of people worldwide every year, leading devastating consequences such as sensory loss, paralysis and bowel/bladder dysfunctions that cost millions of dollars in medical expenses. According to the World Health Organization, although there is no reliable estimation of global prevalence, estimated annual incidence is 40–80 cases per million worldwide. Unfortunately, less than 1% of SCI patients achieve complete neurological recovery by hospital discharge (Spinal Cord Injury (SCI), 2016).

Forthwith after SCI, a rapid activation of glial cells and pericytes occurs, forming a complex healing tissue that is simultaneously beneficial and detrimental for neural repair (Göritz et al., 2011). While in the acute phase macrophages and leukocytes are concentrated in the injury, astrocytes, oligodendrocytes and meningeal cells create a non-permissive biochemical barrier for axonal regrowth at the chronic stage (Rodriguez et al., 2014). Importantly, the early inflammatory responses and the suppression of the astrocytic reactivity increase the extent of damage (Fawcett et al., 2012), a still unknown phenomenon in which the rupture of the blood-spinal cord barrier and the microvascular remodeling in the lesion zone might play key roles (Figley et al., 2014). In this scenario, it is widely accepted that intercommunication between neural and immune cells is crucial to induce reparative responses (Kokaia et al., 2012), with different macrophage populations mediating from neurotoxic to neuro-regenerative responses (Kigerl et al., 2009). In the acute phase, the compromise of the immune system is directly related to the ischemic, necrotic and inflammatory tissue damage typical of SCI (Sapru, 2011). During the chronic phase, there are other pathogenic factors responding to the immune disturbance such as dysregulation mediated by dysautonomia (Garstang and Walker, 2011) or gut dysbiosis (Kigerl et al., 2016).

Nowadays, the cure of SCI remains a major challenge for both researchers and clinicians. There are two main hypotheses for the lack of effective therapies: (a) human spinal motor system relies more dependently on supraspinal control than in other mammals (Capaday, 2002); and (b) SCI produces such state of spinal circuitry depression that diminishes the capacity of these neural networks to generate responses (Harkema, 2008). Other adverse associated factors are the rapid development of inhibitory fibroglial scars and the limited intrinsic ability of the central nervous tissue to regenerate (Fawcett and Asher, 1999; Chen et al., 2002). In this context, research efforts on the development of new materials such as GDMs with ability to initiate and/or mediate suitable reparative responses in the injured neural tissue have a remarkable interest for human health care.

## Graphene-Derived Materials Interfacing The Spinal Cord *In Vivo*

### Studies with Focus on Toxicity and/or Neural Repair

López-Dolado et al. (2015) were first to study the *in vivo* tissue response of the injured rat spinal cord to the implantation of flexible and porous 3D scaffolds composed of reduced graphene oxide (rGO). These scaffolds were fabricated by using the ice segregation-induced self-assembly (ISISA) technique. The lesion model of choice was a right hemisection of approximately 8 mm^3^ at the C6 level, rostral to the bulk of *triceps brachii* motoneurons. This is a suitable model to evaluate therapeutic strategies aimed at promoting neural plasticity and repair. In the main experimental group, rGO scaffolds were placed at the lesion site and covered with a thin gelatin hydrogel film. Animals without injury and those hemisected but not receiving scaffolds served as control groups. In order to study the subacute tissue response to these implants, both locally (at the spinal cord) and systemically (in liver, kidney, lung and spleen), rats were sacrificed at 10 days after surgery. The results revealed that these substrates allowed the formation of a soft interface at the injury site, with no significant differences in the fibroglial scar features with respect to lesions without scaffolds. Due to its porous structure, extracellular matrix molecules (e.g., collagen) and different kinds of cells were able to infiltrate and migrate to the inner parts of the scaffolds contributing to the stabilization of both the scaffold and the lesion site. Colonizing cells were mainly positive for vimentin (indicative of connective tissue cells, glial cells and pericytes, among others) and the β receptor of the platelet-derived growth factor (a regulator of blood vessels formation and early hematopoiesis). In addition, pro-regenerative M2 macrophages were present both at the interface tissue-material and within the scaffold, which could be potentially involved in the initiation of neural repair responses. Finally, neural cell populations were preserved at the perilesional areas and no toxic systemic responses were found, thus indicating the positive biocompatibility of these GDMs with both neural and non-neural tissues once implanted *in vivo*.

**Figure 1:**
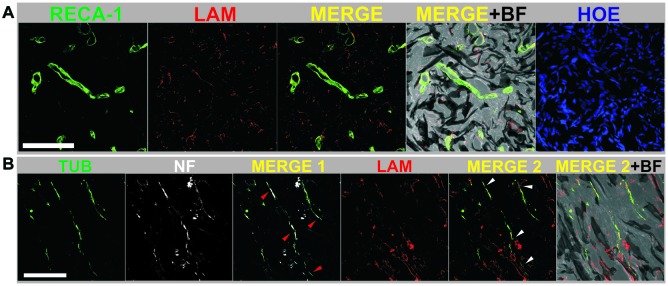
**(A)** Mature blood vessels were detected inside reduced graphene oxide (RGO) scaffolds at 30 days post-injury by labeling RECA-1 (green) and laminin from basal membranes (LAM, red). **(B)** Axons were specifically detected inside RGO scaffolds at 30 days post-injury by expression of β-III tubulin (TUB, green) and neurofilaments (NF, white). Laminin from basal membranes was labeled in red (LAM). Arrow heads indicate areas with coexistence of TUB and NF (red; MERGE 1) and TUB with LAM (white; MERGE 2). Cell nuclei were labeled with Hoechst (HOE). Bright field images (BF) are included to visualize the scaffold structure. Scale bar: 100 μm. Adapted from López-Dolado et al. (2016), Copyright (2016), with permission from Elsevier.

Fueled by these encouraging findings, the authors focused on the tissue responses at 30 days after surgery (early chronic state; López-Dolado et al., 2016). In these studies, it was first confirmed that the scaffolds were properly stabilized, as already achieved at 10 days. As expected from the progression of the lesion, enhanced cell and collagen infiltration within the scaffold was found despite a significant reduction in cells positive for vimentin in comparison to the subacute state. Furthermore, blood vessels, crucial for the initiation of any regenerative processes, increased their size and number over time, both at the interface and, more importantly, inside the graphene substrates. Even though the total amount of macrophages was reduced in comparison with the subacute state, M2 macrophages increased with respect to those with M1-like phenotype (pro-inflammatory). All these changes prompted the formation of a new ambient that allowed the growth of some neuronal axons within the scaffold (not observed at 10 days post-surgery), which were close to functional blood vessels (Figure 1). As found in the subacute state, no evidences of atrophy, inflammation or fibrosis were detected in peripheral organs.

At the brain, Defterali et al. (2016) explored the biocompatibility of rGO and its influence on neurogenesis in the adult mice olfactory bulb (OB) *in vivo*. Specifically, a dispersion of rGO nanosheets in phosphate buffer saline (0.004 μg/μL) was injected in the OB core. By a simultaneous injection of a retroviral vector expressing the enhanced green fluorescent protein, proliferative cells and their progeny were labeled in the subependymal zone/rostral migratory stream of the OB core in the presence of rGO. Major findings revealed that rGO had no deleterious effects either on the survival (measured as number of cells and detection of apoptosis) of the resident populations of neurons and astrocytes or on the newly generated neurons (measured as number of cells). By looking at the number and morphology of microglia cells (Iba1^+^), authors evidenced that alterations on the microglial response at the OB were related to the injection itself rather than the presence of rGO.

Recent studies by Mendonça et al. (2016) focused on the effect of rGO on the blood-brain barrier (BBB) components both *in vitro* and *in vivo*. Under *in vitro* conditions, the exposure to high concentrations of rGO (100 μg/mL) produced a lower toxicity in both rat astrocytes and endothelial cells than rGO functionalized with poly(ethylene glycol) (PEG), which induced a dose- and time-dependent toxicity. For the *in vivo* studies, rGO and rGO-PEG were injected intravenously and their toxic effects on BBB integrity analyzed for different times. Both materials caused a notable downregulation of astrocyte markers (GFAP and connexin-43), endothelial tight (occludin) and adherens (β-catenin) junctions and basal lamina (laminin) at 3 h after administration. Interestingly, this effect disappeared after 7 days of exposure to rGO, while in the rGO-PEG group it was permanent and increased over time. The increase in proteins related to the antioxidant system, such as catalase, in the animals treated with rGO-PEG, accompanied by high levels of intracellular reactive oxygen species, pointed out oxidative stress as one of the main causes of rGO-PEG-mediated damage.

### Studies with Focus on Electro-Magnetic Properties of Graphene

Although more rarely explored, the magnetic properties of GDMs are also attracting attention for their application in neural scenarios. Specifically, magnetic GDMs can generate static magnetic fields (SMFs) that should be taken into account when interfacing the nervous tissue because of their potential influence in cellular intrinsic magnetic fields related to neuronal transmission. As nociception responses depend on this transmission, they could be indirectly influenced by the presence of GDMs. Based on this, Manzo et al. (2017) recently explored the role played by neuronal magnetic fields in nociception by investigating the nociceptive responsiveness to two magnetically-distinct rGO scaffolds (i.e., highly and poorly magnetic). Despite displaying similar chemical features, their respective synthesis processes made them differ in their magnetic behavior. After intrathecal injection in the subarachnoid space of rats, highly magnetic rGO induced more intense nociceptive responses than poorly magnetic one without involving any cytotoxic or inflammatory responses. *In vitro* experiments also demonstrated that the number of KCl-responsive dorsal root ganglion neurons was greater when treated with highly magnetic rGO. Additional studies showed that the activity of both voltage-gated calcium channels and NMDA receptors was altered in the presence of highly magnetic rGO. The disturbance in the neuronal magnetic field increased nociceptive responsiveness, suggesting a role for the magnetic component of the electromagnetic field in neuronal transmission.

### Studies with Focus on Embryogenesis

Boosted by the tremendous development of GDMs in the industry, Chen et al. (2016) pioneered the investigation of environmental risks associated to GO release to the ecosystem by using a zebrafish embryo model. The results evidenced that GO adhered to and enveloped the chorion, blocking pore canals of its membrane and causing subsequent hypoxia and hatching delay. Moreover, GO was able to penetrate the chorion and enter the embryo by endocytosis. Once inside, it damaged mitochondria and translocated to elements of the circulatory system such as the eye, the heart and the yolk sac, with signs of increased oxidative stress, DNA damage, lipid peroxidation and apoptosis. Adverse effects of GO on heart rate and tail/spinal cord flexure were concentration-dependent, evidencing potential risks for embryogenesis in diverse organs including the central nervous system.

In a different study with fertilized chicken eggs *in vivo*, graphene toxicity was studied by inoculating different concentrations of pristine graphene (from 50 μg/L to 10,000 μg/L) dispersed in distilled water (Sawosz et al., 2014). After 19 days of incubation, chicken survival was significantly decreased due to graphene exposure, although neither changes in body and organ weights nor modifications in biochemical indices were found with respect to control embryos. Some atypical electron-dense structures and cellular alterations were noticed in the brain but independent from graphene concentration. According to studies using the zebrafish model described above Chen et al. (2016), results indicate some harmful effects of GDMs in embryogenesis that should be more deeply investigated before advancing on their biomedical application.

## Graphene-Derived Materials Interfacing The Spinal Cord *Ex Vivo* and *In Vitro*

Other outstanding studies in the field used spinal explants as an alternative to *in vivo* murine models. Usmani et al. (2016) pioneered this work by using spinal organotypic slices obtained from spinal cords of E12 C57BL mouse embryos for more than 2 weeks in culture. Particularly, co-cultures of pairs of spinal cord slices separated by a known distance were carried out. The selected materials were 3D scaffolds composed of multi-walled carbon nanotubes (MWCNTs), a different type of carbon-based nanomaterial consisting of concentric layers of graphene rolled up in multi-layered cylinders. A significant development of neuronal projections was found, but with a completely different organization, in both control slices and those interfaced by MWCNT scaffolds. Specifically, the supporting role played by the scaffold made neurites to grow from the explants into a complex net of randomly oriented processes populating the third dimension of the nanoscaffold, whereas they were organized into thick bundles of aligned fibers in controls. Functional tests weakening synaptic inhibition demonstrated slow-paced bursting in all co-cultured explants, with similar inter-event interval in controls and MWCNT scaffolds. However, only a minority of control explants had successful reconnection despite the high number of fiber bundles grown. On the contrary, in the MWCNT scaffold group, a 94% of cases had correlated activity of disinhibited bursting between co-cultured explants. Interestingly, these results could not be reproduced when using a 3D polydimethylsiloxane substrate (although tridimensional, it did not allow the electrical reconnection between explants). Additionally, 3D MWCNT scaffolds did not generate exaggerated tissue responses when implanted in the visual cortex of male rats, as proved by the analysis of the glial scar 4 weeks after surgery and Iba1 levels over time. Finally, the presence of neural cells within the scaffold confirmed that this material could not only integrate into the neural tissue, but also prompt the migration of neuroblasts (localized nearby the implant) to the injury site.

**Figure 2:**
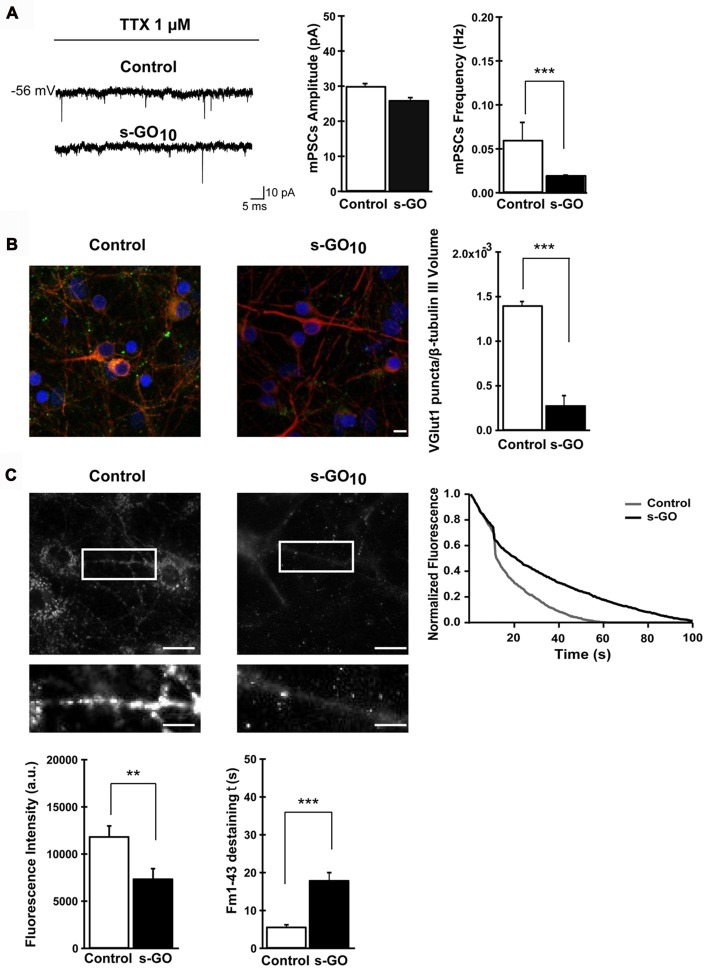
Exposure to small graphene oxide nanosheets (s-GO) at high concentration impaired excitatory synapses. In **(A)**, sample tracings of mPSCs recorded in control and s-GO-treated cultures (left panel) and plots reporting mPSC amplitude and frequency values (right panel) (***p < 0.001, Student’s-*t*-test). In **(B)**, confocal reconstruction of control and s-GO-treated neurons immunolabeled for the vesicular glutamate transporter 1 (VGLUT1; green) and counterstained for cytoskeletal component β tubulin III (red; nuclei are visualized by DAPI labeling in blue; scale bar 10 μm) (***p < 0.001, Student’s-*t*-test). In **(C)**, fluorescence images following staining with FM1–43, control and s-GO-treated (scale bar: 50 μm) (top). The areas in the boxes are higher magnifications to highlight the difference in vesicular staining between the two conditions (scale bar: 10 μm). The plot (top right) reproduces the representative (control and s-GO) traces of FM1–43 de-staining (each trace has been normalized to the maximum fluorescence detected). Bottom: the left plot summarizes the initial raw fluorescent intensities of hippocampal terminals from control and s-GO-treated cultures (**p < 0.01, Mann-Whitney test); the right plot summarizes the decay time constant τ of FM1–43 de-staining in the two conditions (***p < 0.001, Mann-Whitney test). Reprinted with permission from Rauti et al. (2016). Copyright (2016) American Chemical Society.

As the interaction between nanomaterials and cells could drive to intracellular changes due to their association with cell membranes, Rauti et al. (2016) investigated whether the exposure to 2D GO nanosheets could alter cell membrane-based processes, such as vesicle kinetics, and therefore compromise the synaptic communication in both primary hippocampal neurons and cortical glia cultures. Two different kinds of GO nanosheets were used only differing in their lateral dimensions: large and small (l-GO and s-GO, respectively). Interestingly, larger lateral protrusions were more cytotoxic than s-GO, then excluding l-GO samples for the rest of the studies. When focused on s-GO (10 μg/mL), results revealed significant reduction of spontaneous postsynaptic currents (in frequency, not in amplitude). Longer inter-event intervals in Ca^2+^ oscillations and a decrease in vesicular glutamate transporter 1 (VGLUT1) levels were also detected, among other major findings, thus demonstrating interference with neuronal synapses functioning (Figure 2). Importantly, these results were not related to any alteration in cell viability and were not found in cultures exposed to graphene nanosheets.

Pioneer work by Fabbro et al. (2013) explored the use of spinal neurons for evaluating materials aiding to repair lesions at the spinal cord. In these studies, authors tested 2D scaffolds composed of CNTs with dissociated neuronal cultures from neonatal rat spinal cords (P1–P4). Interestingly, the contact with these substrates modulated diverse intracelullar processes including gene translation and transcription. Importantly, neuronal maturation was anticipated by modifying neural ability to generate action potentials, among others. For instance, Alox15 gene expression and its product (related to axon pathfinding) presented higher levels in neurons exposed to CNTs. Nonetheless, authors underlined that a relationship between the cell-material interaction and changes in gene expression patterns could not be directly established.

## Final Conclusions

Thanks to research efforts, knowledge on the interaction of GDMs with central neural cells and tissues is increasing. Extensive results from *in vitro* studies have outlined the relevance that the physic-chemical properties of GDMs play in cell behavior. Some toxicity concerns about their use and the critical influence of particular functionalizations have been also evidenced. In this sense, cellular aspects as diverse as differentiation, neurite outgrowth, oxidative stress and protein and gene expressions could be significantly modulated. The findings from *in vivo* work revealed the ability of GDMs such as graphene, rGO and MWCNTs shaped as 3D scaffolds to initiate beneficial tissue responses when implanted in the injured spinal cord and the brain. Angiogenesis, axon growth, immunomodulatory effects, maintenance of neurogenesis and diminished fibroglial scar formation are some of the most relevant pro-regenerative responses induced by these materials. *Ex vivo* models with spinal cord slices are presented as an attractive and alternative system for testing the phenomena taking place when interfacing central neural tissues with GDMs. Procedures used for testing GDMs in contact with neural tissues and cells should be standardized to diminish contradictory results and facilitate comparisons among laboratories working in the field.

In conclusion, recent advances such as those described in this mini-review are boosting multidisciplinary research in the field that will likely open the path to the exploration of GDMs in translational approaches in biomedicine. Diverse pathologies at the central nervous system including SCI and some brain disorders could eventually benefit from the promising properties of these materials when interfacing the damaged neural tissue. In the next decade, we might assist to a new generation of advanced biomaterials based on graphene with unprecedented modulatory properties in neural systems.

## Author Contributions

All authors equally contributed to the elaboration of this manuscript.

## Conflict of Interest Statement

The authors declare that the research was conducted in the absence of any commercial or financial relationships that could be construed as a potential conflict of interest.
